# Supplementary material for: End Sequence Analysis Toolkit (ESAT) expands the extractable information from single-cell RNA-seq data
Source: Genome Res. 2016 Oct;26(10):1397–410. doi: 10.1101/gr.207902.116 (PMC5052061; doi:10.1101/gr.207902.116)
Supplement: Supplemental Material [file supp_gr.207902.116_Supp_Materials.tar.gz › ESAT-esat_v0.1/doc/html/classbroad_1_1core_1_1datastructures_1_1_interval_tree_3_01_v_01_4.html]

ESAT: broad.core.datastructures.IntervalTree< V > Class Reference


ESAT


- Main Page
- Related Pages
- Packages
- Classes
- Files

- Class List
- Class Index
- Class Hierarchy
- Class Members

All Classes Namespaces Files Functions Variables Enumerator Pages

- broad
- core
- datastructures
- IntervalTree< V >

Classes |
Public Member Functions |
List of all members

broad.core.datastructures.IntervalTree< V > Class Reference

Collaboration diagram for broad.core.datastructures.IntervalTree< V >:

[legend]

|  |  |
| --- | --- |
| Classes | |
| class | FwdIterator |
|  | |
| class | **Node< V1 >** |
|  | |
| class | OverlapIterator |
|  | |
| class | RevIterator |
|  | |
| class | **ValuesIterator< V1 >** |
|  | |

|  |  |
| --- | --- |
| Public Member Functions | |
| int | size () |
|  | |
| boolean | isEmpty () |
|  | |
| void | clear () |
|  | |
| V | put (Interval interval, V value) |
|  | |
| V | put (int start, int end, V value) |
|  | |
| V | placeInTree (int start, int end, V value) |
|  | |
| V | remove (Interval interval) |
|  | |
| V | remove (int start, int end) |
|  | |
| V | remove (int start, int end, V value) |
|  | |
| Node< V > | find (Interval interval) |
|  | |
| Node< V > | find (int start, int end) |
|  | |
| Node< V > | findByIndex (int idx) |
|  | |
| int | getIndex (Interval interval) |
|  | |
| int | getIndex (int start, int end) |
|  | |
| Node< V > | min () |
|  | |
| Node< V > | min (Interval interval) |
|  | |
| Node< V > | min (int start, int end) |
|  | |
| Node< V > | minOverlapper (Interval interval) |
|  | |
| Node< V > | minOverlapper (int start, int end) |
|  | |
| Collection< V > | toCollection () |
|  | |
| List< V > | toList () |
|  | |
| Node< V > | max () |
|  | |
| Node< V > | max (Interval interval) |
|  | |
| Node< V > | max (int start, int end) |
|  | |
| Iterator< Node< V > > | iterator () |
|  | |
| Iterator< V > | valueIterator () |
|  | |
| Iterator< Node< V > > | iterator (Interval interval) |
|  | |
| Iterator< Node< V > > | iterator (int start, int end) |
|  | |
| Iterator< Node< V > > | overlappers (int start, int end) |
|  | |
| Iterator< V > | overlappingValueIterator (int start, int end) |
|  | |
| int | numOverlappers (int start, int end) |
|  | |
| Iterator< Node< V > > | reverseIterator () |
|  | |
| Iterator< Node< V > > | reverseIterator (Interval interval) |
|  | |
| Iterator< Node< V > > | reverseIterator (int start, int end) |
|  | |
| V | getSentinel () |
|  | |
| V | setSentinel (V sentinel) |
|  | |
| void | write (String fileName) throws IOException |
|  | |
| void | write (BufferedWriter bw) throws IOException |
|  | |
| boolean | doesOverlap (int start, int end) |
|  | |

## Detailed Description

A Red-Black tree with intervals for keys. Not thread-safe, and cannot be made so.

Author
:   tsharpe

Version

Revision
:   51146

## Member Function Documentation

|  |  |  |  |  |
| --- | --- | --- | --- | --- |
| void broad.core.datastructures.IntervalTree< V >.clear | ( |  | ) |  |

Remove all entries.

|  |  |  |  |
| --- | --- | --- | --- |
| boolean broad.core.datastructures.IntervalTree< V >.doesOverlap | ( | int | *start*, |
|  |  | int | *end* |
|  | ) |  |  |

|  |  |  |  |  |  |
| --- | --- | --- | --- | --- | --- |
| Node<V> broad.core.datastructures.IntervalTree< V >.find | ( | Interval | *interval* | ) |  |

Find an interval.

Parameters
:   |  |  |
    | --- | --- |
    | interval | The interval sought. |

Returns
:   The Node that represents that interval, or null.

Here is the call graph for this function:

|  |  |  |  |
| --- | --- | --- | --- |
| Node<V> broad.core.datastructures.IntervalTree< V >.find | ( | int | *start*, |
|  |  | int | *end* |
|  | ) |  |  |

Find an interval.

Parameters
:   |  |  |
    | --- | --- |
    | start | The interval's start. |
    | end | The interval's end. |

Returns
:   The Node that represents that interval, or null.

|  |  |  |  |  |  |
| --- | --- | --- | --- | --- | --- |
| Node<V> broad.core.datastructures.IntervalTree< V >.findByIndex | ( | int | *idx* | ) |  |

Find the nth interval in the tree.

Parameters
:   |  |  |
    | --- | --- |
    | idx | The rank of the interval sought (from 0 to size()-1). |

Returns
:   The Node that represents the nth interval.

|  |  |  |  |  |  |
| --- | --- | --- | --- | --- | --- |
| int broad.core.datastructures.IntervalTree< V >.getIndex | ( | Interval | *interval* | ) |  |

Find the rank of the specified interval. If the specified interval is not in the tree, then -1 is returned.

Parameters
:   |  |  |
    | --- | --- |
    | interval | The interval for which the index is sought. |

Returns
:   The rank of that interval, or -1.

Here is the call graph for this function:

|  |  |  |  |
| --- | --- | --- | --- |
| int broad.core.datastructures.IntervalTree< V >.getIndex | ( | int | *start*, |
|  |  | int | *end* |
|  | ) |  |  |

Find the rank of the specified interval. If the specified interval is not in the tree, then -1 is returned.

Parameters
:   |  |  |
    | --- | --- |
    | start | The interval's start. |
    | end | The interval's end. |

Returns
:   The rank of that interval, or -1.

|  |  |  |  |  |
| --- | --- | --- | --- | --- |
| V broad.core.datastructures.IntervalTree< V >.getSentinel | ( |  | ) |  |

Get the special sentinel value that will be used to signal novelty when putting a new interval into the tree, or to signal "not found" when removing an interval. This is null by default.

Returns
:   The sentinel value.

|  |  |  |  |  |
| --- | --- | --- | --- | --- |
| boolean broad.core.datastructures.IntervalTree< V >.isEmpty | ( |  | ) |  |

|  |  |  |  |  |
| --- | --- | --- | --- | --- |
| Iterator<Node<V> > broad.core.datastructures.IntervalTree< V >.iterator | ( |  | ) |  |

Return an iterator over the entire tree.

Returns
:   An iterator.

|  |  |  |  |  |  |
| --- | --- | --- | --- | --- | --- |
| Iterator<Node<V> > broad.core.datastructures.IntervalTree< V >.iterator | ( | Interval | *interval* | ) |  |

Return an iterator over all intervals greater than or equal to the specified interval.

Parameters
:   |  |  |
    | --- | --- |
    | interval | The minimum interval. |

Returns
:   An iterator.

Here is the call graph for this function:

|  |  |  |  |
| --- | --- | --- | --- |
| Iterator<Node<V> > broad.core.datastructures.IntervalTree< V >.iterator | ( | int | *start*, |
|  |  | int | *end* |
|  | ) |  |  |

Return an iterator over all intervals greater than or equal to the specified interval.

Parameters
:   |  |  |
    | --- | --- |
    | start | The interval's start. |
    | end | The interval's end. |

Returns
:   An iterator.

|  |  |  |  |  |
| --- | --- | --- | --- | --- |
| Node<V> broad.core.datastructures.IntervalTree< V >.max | ( |  | ) |  |

Find the greatest interval in the tree.

Returns
:   The latest interval, or null if the tree is empty.

|  |  |  |  |  |  |
| --- | --- | --- | --- | --- | --- |
| Node<V> broad.core.datastructures.IntervalTree< V >.max | ( | Interval | *interval* | ) |  |

Find the latest interval in the tree less than or equal to the specified interval.

Parameters
:   |  |  |
    | --- | --- |
    | interval | The interval sought. |

Returns
:   The latest <= interval, or null if there is none.

Here is the call graph for this function:

|  |  |  |  |
| --- | --- | --- | --- |
| Node<V> broad.core.datastructures.IntervalTree< V >.max | ( | int | *start*, |
|  |  | int | *end* |
|  | ) |  |  |

Find the latest interval in the tree less than or equal to the specified interval.

Parameters
:   |  |  |
    | --- | --- |
    | start | The interval's start. |
    | end | The interval's end. |

Returns
:   The latest >= interval, or null if there is none.

|  |  |  |  |  |
| --- | --- | --- | --- | --- |
| Node<V> broad.core.datastructures.IntervalTree< V >.min | ( |  | ) |  |

Find the least interval in the tree.

Returns
:   The earliest interval, or null if the tree is empty.

|  |  |  |  |  |  |
| --- | --- | --- | --- | --- | --- |
| Node<V> broad.core.datastructures.IntervalTree< V >.min | ( | Interval | *interval* | ) |  |

Find the earliest interval in the tree greater than or equal to the specified interval.

Parameters
:   |  |  |
    | --- | --- |
    | interval | The interval sought. |

Returns
:   The earliest >= interval, or null if there is none.

Here is the call graph for this function:

|  |  |  |  |
| --- | --- | --- | --- |
| Node<V> broad.core.datastructures.IntervalTree< V >.min | ( | int | *start*, |
|  |  | int | *end* |
|  | ) |  |  |

Find the earliest interval in the tree greater than or equal to the specified interval.

Parameters
:   |  |  |
    | --- | --- |
    | start | The interval's start. |
    | end | The interval's end. |

Returns
:   The earliest >= interval, or null if there is none.

|  |  |  |  |  |  |
| --- | --- | --- | --- | --- | --- |
| Node<V> broad.core.datastructures.IntervalTree< V >.minOverlapper | ( | Interval | *interval* | ) |  |

Find the earliest interval in the tree that overlaps the specified interval.

Parameters
:   |  |  |
    | --- | --- |
    | interval | The interval sought. |

Returns
:   The earliest overlapping interval, or null if there is none.

Here is the call graph for this function:

|  |  |  |  |
| --- | --- | --- | --- |
| Node<V> broad.core.datastructures.IntervalTree< V >.minOverlapper | ( | int | *start*, |
|  |  | int | *end* |
|  | ) |  |  |

Find the earliest interval in the tree that overlaps the specified interval.

Parameters
:   |  |  |
    | --- | --- |
    | start | The interval's start. |
    | end | The interval's end. |

Returns
:   The earliest overlapping interval, or null if there is none.

|  |  |  |  |
| --- | --- | --- | --- |
| int broad.core.datastructures.IntervalTree< V >.numOverlappers | ( | int | *start*, |
|  |  | int | *end* |
|  | ) |  |  |

|  |  |  |  |
| --- | --- | --- | --- |
| Iterator<Node<V> > broad.core.datastructures.IntervalTree< V >.overlappers | ( | int | *start*, |
|  |  | int | *end* |
|  | ) |  |  |

Return an iterator over all intervals overlapping the specified range.

Parameters
:   |  |  |
    | --- | --- |
    | start | The range start. |
    | end | The range end. |

Returns
:   An iterator.

|  |  |  |  |
| --- | --- | --- | --- |
| Iterator<V> broad.core.datastructures.IntervalTree< V >.overlappingValueIterator | ( | int | *start*, |
|  |  | int | *end* |
|  | ) |  |  |

|  |  |  |  |
| --- | --- | --- | --- |
| V broad.core.datastructures.IntervalTree< V >.placeInTree | ( | int | *start*, |
|  |  | int | *end*, |
|  |  | V | *value* |
|  | ) |  |  |

Put a new interval into the tree (or update the value associated with an existing interval). If the interval is novel, the special sentinel value is returned.

Parameters
:   |  |  |
    | --- | --- |
    | start | The interval's start. |
    | end | The interval's end. |
    | value | The associated value. |

Returns
:   The old value associated with that interval, or the sentinel.

|  |  |  |  |
| --- | --- | --- | --- |
| V broad.core.datastructures.IntervalTree< V >.put | ( | Interval | *interval*, |
|  |  | V | *value* |
|  | ) |  |  |

Put a new interval into the tree (or update the value associated with an existing interval). If the interval is novel, the special sentinel value is returned.

Parameters
:   |  |  |
    | --- | --- |
    | interval | The interval. |
    | value | The associated value. |

Returns
:   The old value associated with that interval, or the sentinel.

Here is the call graph for this function:

|  |  |  |  |
| --- | --- | --- | --- |
| V broad.core.datastructures.IntervalTree< V >.put | ( | int | *start*, |
|  |  | int | *end*, |
|  |  | V | *value* |
|  | ) |  |  |

Rewrote put to check that there is an already existing node

|  |  |  |  |  |  |
| --- | --- | --- | --- | --- | --- |
| V broad.core.datastructures.IntervalTree< V >.remove | ( | Interval | *interval* | ) |  |

Remove an interval from the tree. If the interval does not exist in the tree the special sentinel value is returned.

Parameters
:   |  |  |
    | --- | --- |
    | interval | The interval to remove. |

Returns
:   The value associated with that interval, or the sentinel.

|  |  |  |  |
| --- | --- | --- | --- |
| V broad.core.datastructures.IntervalTree< V >.remove | ( | int | *start*, |
|  |  | int | *end* |
|  | ) |  |  |

Remove an interval from the tree. If the interval does not exist in the tree the special sentinel value is returned.

Parameters
:   |  |  |
    | --- | --- |
    | start | The interval's start. |
    | end | The interval's end. |

Returns
:   The value associated with that interval, or the sentinel.

|  |  |  |  |
| --- | --- | --- | --- |
| V broad.core.datastructures.IntervalTree< V >.remove | ( | int | *start*, |
|  |  | int | *end*, |
|  |  | V | *value* |
|  | ) |  |  |

Remove a specific value from this position

Parameters
:   |  |  |
    | --- | --- |
    | start |  |
    | end |  |
    | value |  |

Returns

|  |  |  |  |  |
| --- | --- | --- | --- | --- |
| Iterator<Node<V> > broad.core.datastructures.IntervalTree< V >.reverseIterator | ( |  | ) |  |

Return an iterator over the entire tree that returns intervals in reverse order.

Returns
:   An iterator.

|  |  |  |  |  |  |
| --- | --- | --- | --- | --- | --- |
| Iterator<Node<V> > broad.core.datastructures.IntervalTree< V >.reverseIterator | ( | Interval | *interval* | ) |  |

Return an iterator over all intervals less than or equal to the specified interval, in reverse order.

Parameters
:   |  |  |
    | --- | --- |
    | interval | The maximum interval. |

Returns
:   An iterator.

Here is the call graph for this function:

|  |  |  |  |
| --- | --- | --- | --- |
| Iterator<Node<V> > broad.core.datastructures.IntervalTree< V >.reverseIterator | ( | int | *start*, |
|  |  | int | *end* |
|  | ) |  |  |

Return an iterator over all intervals less than or equal to the specified interval, in reverse order.

Parameters
:   |  |  |
    | --- | --- |
    | start | The interval's start. |
    | end | The interval's end. |

Returns
:   An iterator.

|  |  |  |  |  |  |
| --- | --- | --- | --- | --- | --- |
| V broad.core.datastructures.IntervalTree< V >.setSentinel | ( | V | *sentinel* | ) |  |

Set the special sentinel value that will be used to signal novelty when putting a new interval into the tree, or to signal "not found" when removing an interval.

Parameters
:   |  |  |
    | --- | --- |
    | sentinel | The new sentinel value. |

Returns
:   The old sentinel value.

|  |  |  |  |  |
| --- | --- | --- | --- | --- |
| int broad.core.datastructures.IntervalTree< V >.size | ( |  | ) |  |

Return the number of intervals in the tree.

Returns
:   The number of intervals.

|  |  |  |  |  |
| --- | --- | --- | --- | --- |
| Collection<V> broad.core.datastructures.IntervalTree< V >.toCollection | ( |  | ) |  |

|  |  |  |  |  |
| --- | --- | --- | --- | --- |
| List<V> broad.core.datastructures.IntervalTree< V >.toList | ( |  | ) |  |

|  |  |  |  |  |
| --- | --- | --- | --- | --- |
| Iterator<V> broad.core.datastructures.IntervalTree< V >.valueIterator | ( |  | ) |  |

Return an iterator over the entire tree values

Returns
:   An iterator

|  |  |  |  |  |  |
| --- | --- | --- | --- | --- | --- |
| void broad.core.datastructures.IntervalTree< V >.write | ( | String | *fileName* | ) | throws IOException |

|  |  |  |  |  |  |
| --- | --- | --- | --- | --- | --- |
| void broad.core.datastructures.IntervalTree< V >.write | ( | BufferedWriter | *bw* | ) | throws IOException |

---

The documentation for this class was generated from the following file:

- java/broad/core/datastructures/IntervalTree.java


---

Generated on Fri Aug 15 2014 15:10:51 for ESAT by  

 1.8.7
